# Grain Boundary Induced Bias Instability in Soluble Acene-Based Thin-Film Transistors

**DOI:** 10.1038/srep33224

**Published:** 2016-09-12

**Authors:** Ky V. Nguyen, Marcia M. Payne, John E. Anthony, Jung Hun Lee, Eunjoo Song, Boseok Kang, Kilwon Cho, Wi Hyoung Lee

**Affiliations:** 1 Department of Organic and Nano System Engineering, Konkuk University, Seoul 05029, Korea; 2 Department of Chemistry, University of Kentucky, Lexington 40506, USA; 3 Department of Chemical Engineering, Pohang University of Science and Technology, Pohang 37673, Korea

## Abstract

Since the grain boundaries (GBs) within the semiconductor layer of organic field-effect transistors (OFETs) have a strong influence on device performance, a substantial number of studies have been devoted to controlling the crystallization characteristics of organic semiconductors. We studied the intrinsic effects of GBs within 5,11-bis(triethylsilylethynyl) anthradithiophene (TES-ADT) thin films on the electrical properties of OFETs. The GB density was easily changed by controlling nulceation event in TES-ADT thin films. When the mixing time was increased, the number of aggregates in as-spun TES-ADT thin films were increased and subsequent exposure of the films to 1,2-dichloroethane vapor led to a significant increase in the number of nuleation sites, thereby increasing the GB density of TES-ADT spherulites. The density of GBs strongly influences the angular spread and crystallographic orientation of TES-ADT spherulites. Accordingly, the FETs with higher GB densities showed much poorer electrical characteristics than devices with lower GB density. Especially, GBs provide charge trapping sites which are responsible for bias-stress driven electrical instability. Dielectric surface treatment with a polystyrene brush layer clarified the GB-induced charge trapping by reducing charge trapping at the semiconductor-dielectric interface. Our study provides an understanding on GB induced bias instability for the development of high performance OFETs.

Organic field-effect transistors (OFETs) have received much attention due to their potential applications in light-weight, low-cost, flexible electronics such as radio-frequency identification (RFID) tags, display drivers and sensors^1^,^2^,^3^. However, overall OFET performance is still too low for commercialization and is strongly dependent on the microstructure of the organic component. Organic single crystals free of defects and grain boundaries (GBs) have shown good electrical properties such as high field-effect mobility and excellent gate bias stability^4^,^5^,^6^,^7^,^8^,^9^. These superior performances aside, such high purity single crystals can only be grown in well-controlled conditions with special techniques^10^. Typically, thermally-evaporated organic semiconductor films (e.g., pentacene film) show a polycrystalline nature consisting of individual grains and GBs^11^,^12^. Kelvin probe microscopy studies have successfully proved charge trapping at the GBs of pentacene film^13^. However, a decrease of GBs in pentacene film does not directly lead to an increase of field-effect mobility. It is thought that an in-plane herringbone packing motif and polymorphism (i.e., thin-film phase, bulk phase) increase the complexity of the structure of polycrystalline pentacene films^12^,^14^,^15^. For this reason, inhomogeneous charge trapping, regardless of GBs, was reported by several research groups that examined pentacene films^16^.

Solution-processed small molecular organic semiconductors (e.g., so-called, soluble acenes) were designed both for enabling solution processability and enhancing charge transport properties^17^,^18^,^19^,^20^,^21^,^22^,^23^,^24^,^25^,^26^,^27^. This was accomplished by attaching bulk side groups to pentacene or anthradithiophene backbones. 6,13-bis(triisopropylsilylethynyl) pentacene (TIPS-PEN), 5,11-bis(triethylsilylethynyl) anthradithiophene (TES-ADT), and 2,8-difluoro-5,11-bis(triethylsilylethynyl) anthradithiophene (F-TESADT) are typical examples^23^,^24^,^25^. In addition to the increased solubility, these materials adopt cofacial in-plane stacking with reduced π-π stacking distances. In one report, the field-effect mobility of FETs with solution sheared TIPS-PEN film suggest mobility over 4.6 cm^2^/V-s, proving the outstanding charge transport properties of these materials^28^. Also, TES-ADT film could be crystallized easily with minimal solvent vapor treatment^29^,^30^,^31^,^32^,^33^,^34^,^35^,^36^,^37^,^38^,^39^,^40^,^41^,^42^, so that spherulites with a size of a few mm were easily grown and excellent electrical properties were obtained with solvent vapor annealed TES-ADT films. The TES-ADT spherulite is an interesting example with which to study GB effects in organic semiconductors as the in-plane packing motif of TES-ADT is much simpler than that of pentacene film^25^. To this end, a small amount of F-TESADT added to a TES-ADT solution and heterogeneous nucleation in TES-ADT (host)/F-TESADT (guest) film leads to an increase in the number of GBs in TES-ADT spherulites during solvent vapor treatment^41^,^42^. This approach is extremely beneficial for controlling grain size (or number of GBs) of TES-ADT spherulites. However, the host-guest system fails to exhibit the intrinsic effects of GBs because guest molecules (here, F-TESADT), as well as GBs, can also be charge trapping sites. GBs between crystallites can create a number of traps which cause degradation of device characteristics such as threshold voltage shifts and on-current changes (bias-stress instability) upon prolonged gate bias, which will disrupt the functioning of the display pixels connected to OFETs^43^. Therefore, the rational strategy for reducing these traps and the electrical instability is to control the density of GBs.

In this study, we examined intrinsic effects of GBs on the electrical properties of TES-ADT spherulites. Only a single TES-ADT component was used for preparing the solution while the mixing time was varied from 5 mins to 12 hours. As solution mixing time increased, self-aggregation of TES-ADT molecules occurred and a spin-cast film showed many aggregates. This in turn resulted in a decrease in the grain size of TES-ADT spherulites (or an increase in the number of GBs) during solvent vapor annealing. Correlations between solution mixing time and the spherulite size of TES-ADT films after solvent vapor annealing were studied. In addition, the effects of GBs in TES-ADT spherulites on electrical properties (i.e., charge trapping, field-effect mobility, and gate bias stability) of TES-ADT FETs were examined by employing TES-ADT spherulites in the active layer of OFETs. Furthermore, a very thin organic layer was introduced as a surface modifier of the SiO_2_ dielectric for reducing charge trapping at the semiconductor-dielectric interface and thereby clarifying the GB-induced charge trapping.

## Results and Discussion

### Morphology of TES-ADT thin films

**Figure 1:**
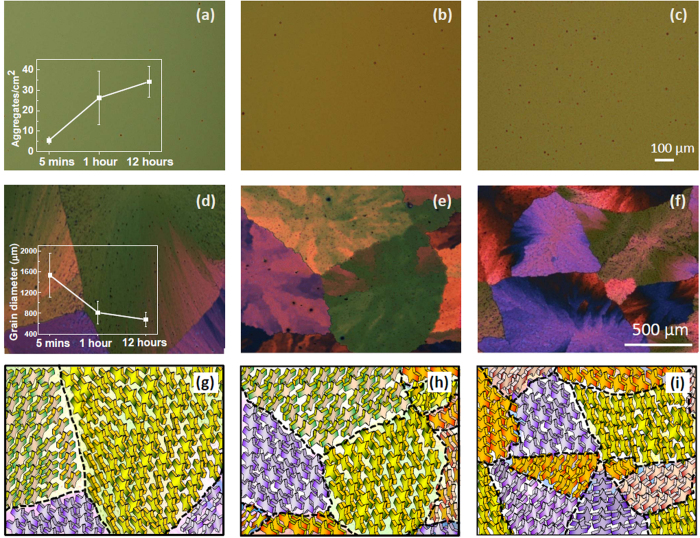
Optical micrographs of TES-ADT films whose solutions were prepared for 5 minutes, 1 hour and 12 hours of mixing before solvent vapor annealing (**a**–**c**). Polarized optical micrographs of these TES-ADT films after solvent vapor annealing (**d**–**f**): (**d**) 5 minutes, (**e**) 1 hour, (**f**) 12 hours. The insets in (**a**,**d**) represent variations in number of aggregates per square centimeter and average grain diameter (μm), respectively. Schemes showing molecular arragements of TES-ADT films with different GB densities: (**g**) 5 minutes, (**h**) 1 hour, (**i**) 12 hours.

To understand spherulitic growth in TES-ADT thin films, the film morphology before and after solvent vapor annealing were first studied with an optical microscope. Before solvent vapor annealing, TES-ADT thin films were examined with an optical microscope when varying the mixing time from 5 minutes to 12 hours. Figure 1a–f display optical microscopy images of TES-ADT films of three samples before and after the solvent-vapor annealing process. Since toluene is a good solvent for TES-ADT, there is no observed feature in the TES-ADT film after spin coating, indicating that the as-spun TES-ADT forms largely amorphous films. Interestingly, after increasing the mixing time from 5 mins to 1 hour and then 12 hours, a number of small aggregates occurred (Fig. 1b,c), which indicates that TES-ADT molecules in solution could be clustered together to form aggregates. Figure 1a inset shows gradual increase in the number of aggregates with mixing time.

Exposing the samples to DCE vapor led to a significant change in the optical properties of the films. Figure 1d–f show polarized optical microscopy images of TES-ADT films after they were exposed to DCE vapor for from 2 to 4 minutes. We can observe different spherulite sizes among the three samples (Fig. 1d inset). The sizes of the spherulites were found to range from several hundred micrometers to a few millimeters. To be more specific, the average grain size of samples mixed for 12 hours is around 600 μm, which is the smallest compared to that of samples mixed for 1 hour (grain size is around 800 μm) and samples mixed for 5 minutes (grain size is around 1.5 mm, even up to 2 mm).

The observed grain size differences in our experiments can be understood by considering the crystallization condition of the organic semiconductors. We suggest that aggregates observed when increasing the mixing time provided a large number of nucleation sites, resulting in the growth of small TES-ADT grains. We speculate that aggregates in TES-ADT film induce surface irregularity, thereby accelerating nucleation events. Because SiO_2_ substrates were cleaned carefully for avoiding contamination, and because no additives were used in TES-ADT solution, other factors affecting nucleation behaviors could be ignored. This crystallization behavior of TES-ADT is quite different from the previously reported results that increased nucleation event by using roughness of the substrate (PMMA residue) and guest molecules (F-TESADT)^36^,^41^,^42^.

**Figure 2:**
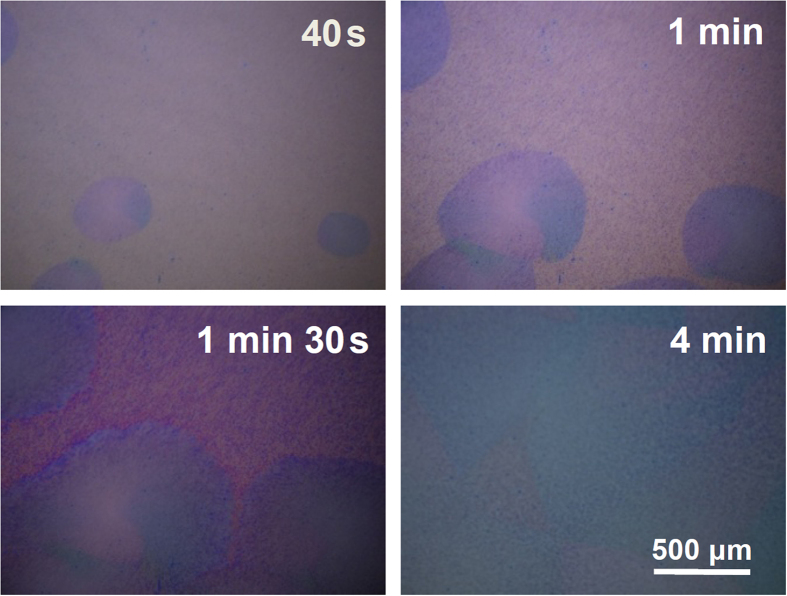
Optical microscopy images of *in-situ* spherulite growth in a spun-cast TES-ADT film (12 hours of mixing) during exposure to DCE vapor for 40 s, 1 min, 1 min 30 s and 4 mins.

Figure 2 shows optical microscopy images of spherulite growth in a spun-cast TES-ADT film during exposure to DCE vapor for 40 seconds, 60 seconds, 90 seconds, and 4 minutes. As seen from the figure, after 40 seconds of DCE vapor exposure, spherulites growing radically from their nucleation centers are clearly visible. In addition, these spherulites become large when the solvent exposure time increases. After 4 minutes of the solvent exposure, the TES-ADT films were totally crystallized in their entireties. It has been shown that the growth of each spherulite develops radically from its nucleation center and that the crystallization does not end until the spherulites impinge upon one another^34^,^39^. From this analysis, spherulite size is determined by the number of nucleation sites at the early stage of the nucleation event. In our experiments, the low number of nucleation sites in TES-ADT thin film prepared from mixing 5 minutes results in the biggest spherulite size after crystallization with DCE vapor. Figure 1g–i show schematic representations showing molecular arrangements of TES-ADT with different GB densities. It is expected that molecular orientation of TES-ADT will be changed according to the GB densities as discussed in the following section.

### Crystal structure of TES-ADT thin films

**Figure 3:**
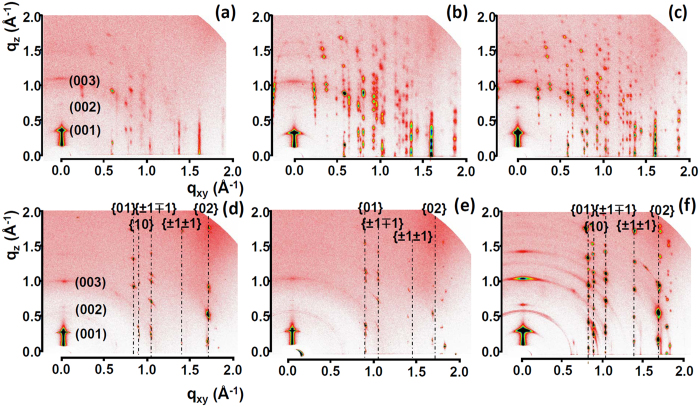
2D-GIXD patterns of as-spun TES-ADT thin films (**a**–**c**) and solvent-vapor annealed TES-ADT thin films (**d**–**f**) whose solutions were prepared for 5 minutes (**a**,**d**), 1 hour (**b**,**e**) and 12 hours of mixing (**c**,**f**), respectively.

To examine the structural changes of the TES-ADT films after solvent vapor annealing, X-ray diffraction measurements were carried out. Two dimensional grazing incidence X-ray diffraction (2D-GIXD) analysis produced detailed information about the crystalline orientation along the out-of-plane and in-plane directions. Before solvent annealing, the observation from optical micrographs of as-spun TES-ADT made us accept that as-spun TES-ADT thin films are amorphous. From 2D-GIXD measurements, a large number of reflection spots have been however found, indicating that a limited arrangement of TES-ADT molecules does still exist in as-spun TES-ADT thin films. As shown in Fig. 3a–c, there existed (001), (002), and (003) reflection spots and their intensities increased with mixing time, which indicates that TES-ADT molecules crystallize with silyl groups on the substrate surface. The number of reflection spots in the TES-ADT film from mixing for 5 minutes was small and their intensities were weak in comparison with those of samples with mixing for 1 hour and mixing for 12 hours, indicating low crystallinity along the in-plane direction^44^. The number of reflection spots in TES-ADT from mixing for 12 hours is the largest, indicating that the TES-ADT thin film contains many crystal domains with different crystalline orientations because of aggregation-induced crystallization. However, these diffraction patterns actually did not match with crystallographic information in TES-ADT. We speculate that as-spun TES-ADT thin film adopts a polymorphic crystal form and thus molecular arrangement along the in-plane direction is complex. Notwithstanding these difficulties, we can say that there is an aggregation-induced crystallization for the TES-ADT film from long mixing times.

After solvent annealing, the as-spun TES-ADT thin films were converted to be fully crystalline as shown in the polarized optical micrographs (Fig. 1d–f). 2D-GIXD diffraction patterns on solvent-vapor annealed TES-ADT as illustrated in Fig. 3d–f are totally different from those of TES-ADT films before solvent vapor annealing. By analyzing these reflection patterns, we are able to identify the best-fit lattice parameters (lengths a, b, c and angles α, β, γ) for the unit cell of the solvent-vapor annealed TES-ADT thin films. From Fig. 3d–f, the (001), (002), and (003) reflection spots in the out-of-plane direction (along q_z_) are regularly arranged at intervals of 0.37 Å^−1^, corresponding to a d-spacing of 16.9 Å which indicates that TES-ADT molecules crystallize with silyl groups on the substrate surface. These reflection spots are well matched with reference crystallographic information of TES-ADT powder diffraction and solvent-vapor annealed TES-ADT films^45^. When mixing time was increased, solvent-vapor annealed TES-ADT thin films were composed of many small grains with high density of GBs. For this reason, the TES-ADT crystals have different crystallographic orientations. Accordingly, angular spreads of the spots in the 12-hour sample are large, indicating that its film contains crystals with tilted orientations. In addition, many in-plane reflection spots such as {10} reflections are visible, implying that the 12-hour sample contains many crystal domains with different crystallographic orientations^44^. This is due to the existence of GB as schematically drawn in Fig. 1i. On the other hand, the 5-min sample consists of nearly a single grain with a negligible density of GBs because TES-ADT molecules are crystallized from one nucleation point. Thus, in-plane reflections exhibited low values of angular spreads (Fig. 1g). TES-ADT molecules in the 5-min sample have well-ordered 3-dimensional multi-layered stacking, which is extremely beneficial for the maximization of π-orbital overlaps for lateral charge transport.

The mechanism for TES-ADT crystal growth that happens during solvent vapor annealing was proposed by Lee *et al*.^39^. Toluene in the TES-ADT solution rapidly evaporated during spin casting of the TES-ADT solution. This, therefore, made TES-ADT molecules difficult to crystallize and led to a low crystalline film as a consequence. When exposing the as-spun TES-ADT film to DCE vapor, DCE molecules penetrated into the TES-ADT film giving TES-ADT molecules the ability to be changed into an energetically stable structure. Since the (001) surface including the silyl groups has the lowest surface energy in the TES-ADT molecule, and crystals tend to orient in such a way that the plane with the lowest surface energy is parallel to the substrate, the TES-ADT molecules crystallize with silyl groups on the substrate surface^39^,^46^. Then intermolecular π-π stacking along in-plane direction was maximized and spherulites were formed as a result of DCE vapor annealing.

### Electrical characteristics of TES-ADT FETs

**Figure 4:**
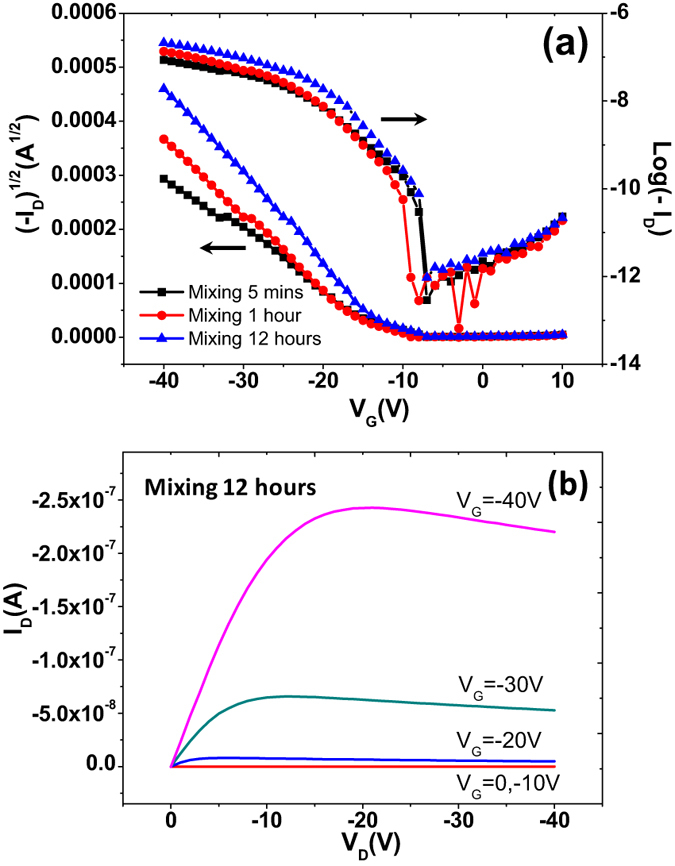
Typical transfer (**a**) and output (**b**) characteristics of TES-ADT transistors based on an untreated SiO_2_ dielectric before solvent vapor annealing.

After depositing source and drain electrodes onto as-spun TES-ADT films, current voltage characteristics were measured. The transfer characteristics with a sweep voltage V_G_ from 10 V to −40 V and a step of −1 V are shown in Fig. 4a. On-current levels of devices clearly increase with increased mixing time, indicating that mixing in oxygen-free/dark condition neither induces degradation of the TES-ADT molecule nor generates impurities. Instead, mixing-induced aggregations of TES-ADT molecules are beneficial for the enhancement of electrical performance in TES-ADT transistors. The average field-effect mobility shown in Table 1 increased from 0.22 × 10^−2 ^cm^2^/V-s (5-min sample) to 0.34 × 10^−2 ^cm^2^/V-s (1-hour sample) and reached the highest value of 0.39 × 10^−2 ^cm^2^/V-s (12-hour sample). The relatively low mobilities can be attributed to low crystallinity in as-prepared TES-ADT films. Although there exists an arrangement of TES-ADT molecules along the out-of-plane direction right after spin coating, π-π stacking among neighboring TES-ADT molecules is not maximized. As a result, charge transport along the lateral direction is not facilitated, thereby leading to low field-effect mobility^29^,^39^,^46^. The increase in field-effect mobility with increasing mixing time can be explained by considering aggregation-induced crystallization (Figs 1 and 3). The TES-ADT solution mixed longer leads to aggregation of the TES-ADT molecules where π-orbital overlap among the TES-ADT molecules is enhanced. Therefore, mixing the sample for 12 hours exhibits the highest mobility compared to the two remaining samples.

**Figure 5:**
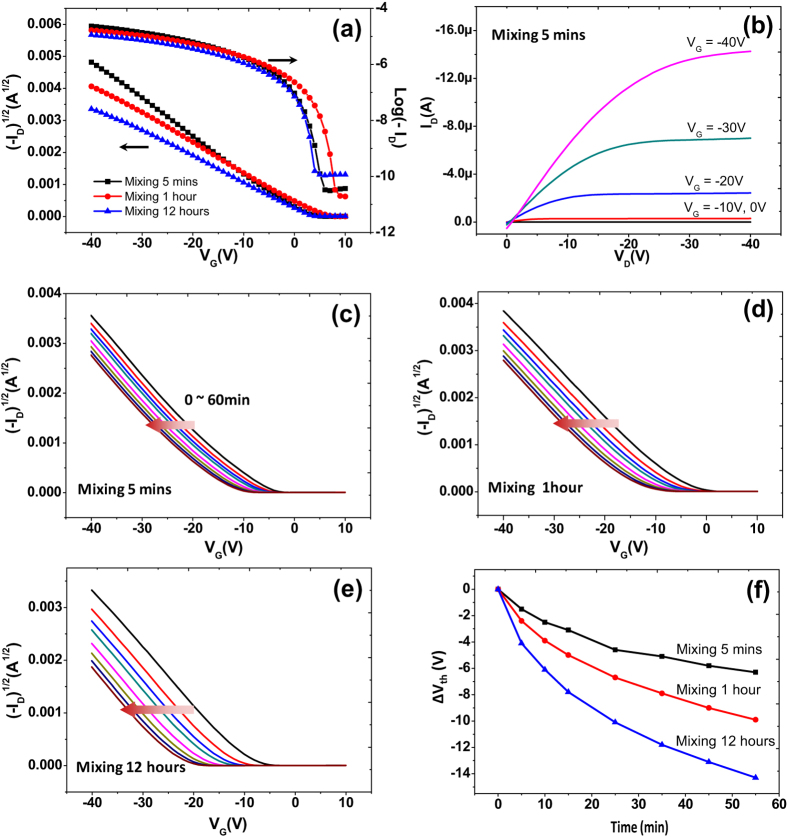
Typical transfer (**a**) and output (**b**) characteristics of TES-ADT FETs based on an untreated SiO_2_ dielectric after solvent vapor annealing. I_D_−V_G_ transfer curves of TES-ADT FETs based on an untreated SiO_2_ dielectric as a function of stress time, under an applied gate bias stress of −40 V (V_D_ = −5 V) (**c**–**e**), and relative threshold voltage shift ΔV_th_ as a function of stress time (**f**).

After solvent vapor annealing, TES-ADT films showed enhanced crystallinity as shown in Fig. 3. High crystallinity in organic semiconductor films is typically linked to high electrical performances of OFETs. Grain sizes or grain boundaries also play an important role in the electrical performance. In this regard, comparing the charge transport characteristics of TES-ADT spherulites with different grain sizes is an interesting topic. There is a very dramatic improvement in the device characteristics of TES-ADT FETs after solvent vapor annealing, compared to the results before solvent vapor annealing. As shown in Fig. 5a, on-currents increased by at least a factor of 100. As can be seen from Table 1, the average field-effect mobility of the 5-min sample was 0.248 cm^2^/V-s, increased by a factor of 100 compared to 5-min samples before vapor annealing. The 1-hour samples and 12-hour samples also experienced a similar trend. Threshold voltages (V_th_) of all three samples are positive values and are close to zero. The current on-off ratio of 10^5^ with the 12-hour sample is the same as with the 12-hour sample before solvent annealing. The improvements in field-effect mobility are closely correlated with high crystallinity in the TES-ADT film (Fig. 3), and matched well with the previous results of Dickey *et al*.^29^ and Lee *et al*.^39^.

Figure S1 in Supporting information shows the different GB density in the channel regions among the three samples. There is an increase in GB density from mixing for 5 mins to mixing for 12 hours. The drain current levels in Fig. 5a show an obvious decrease with increasing numbers of grain boundaries in the channel areas. From Table 1, there is an apparent tendency toward the degradation of mobility with decreasing grain size (or increase in the number of GBs). The explanation for this interesting finding can be found in the structural characteristics of GBs. GBs are disordered regions that act as charge trapping sites. These trapping sites prevent charge carriers from being transported along the channel regions, thereby leading to low mobility. In addition, the 12-hour sample contains misoriented crystals with various crystallographic orientations. This also contributes to the reduced electrical performances observed in FETs of the 12-hour sample. The recent work by Lee *et al*. examined the dependence of the field-effect mobility of FETs on the grain size of TES-ADT. By adding additives, FTES-ADT with concentrations ranging from 1.17 mol% to 0.03 mol%, the grain size of TES-ADT was changed from 30 μm to 2700 μm and the resulting mobility rose from 0.005 cm^2^/V-s to 0.36 cm^2^/V-s^42^. Their results are related to reduced charge trapping at the GBs. However, in their system, GBs contain heterogeneous FTES-ADT molecules. These molecules themselves could be charge trapping sites.

The long-term operational stability of TES-ADT FETs was examined by monitoring V_th_ as a function of time under an applied gate-bias stress of −40 V for 1 hour under vacuum condition. The V_th_ was found to shift in the direction of the applied gate-bias stress, that is, to more negative voltages for an applied negative gate voltage or to more positive voltages for an applied positive gate voltage^43^,^47^,^48^,^49^,^50^. That can be due to charge trapping at the semiconducting layer, dielectric layer, semiconductor-dielectric interface, and/or semiconductor-electrode contacts. To be more specific, amorphous regions and GBs within organic semiconductor films could be origins for charge trapping in the semiconductor^33^,^43^. Figure 5c–e represents the electrical stabilities of TES-ADT FETs for all three samples. The V_th_ shift was much smaller in the 5-min sample than in the 1-hour and 12-hour samples. The V_th_ shift was the largest in the 12-hour sample. The relative threshold voltage shifts (ΔV_th_) of the TES-ADT FETs are summarized in Fig. 5f. The large V_th_ shift observed with the 12-hour device is due to hole trapping at GBs. When there is a continuous negative gate bias, holes are trapped in GBs thereby resulting in a shift of transfer curves to the negative gate-bias direction. Quantitative information (τ and β) was extracted by fitting curves in Fig. 5f with the stretched exponential equation^43^. Calculated characteristic time (τ) and dispersion parameter (β) were shown in Table 2. The value of τ decreased with increasing GB density while the value of β did not change much. Because τ is closely related to the mean time of charge carriers to be in the mobile state, low τ value in 12 hours sample is directly linked to charge trapping at the GBs. Charge trappings at GBs were previously reported in several reports, which used pentacene or TES-ADT as a semiconducting layer^13^,^37^,^51^,^52^.

**Figure 6:**
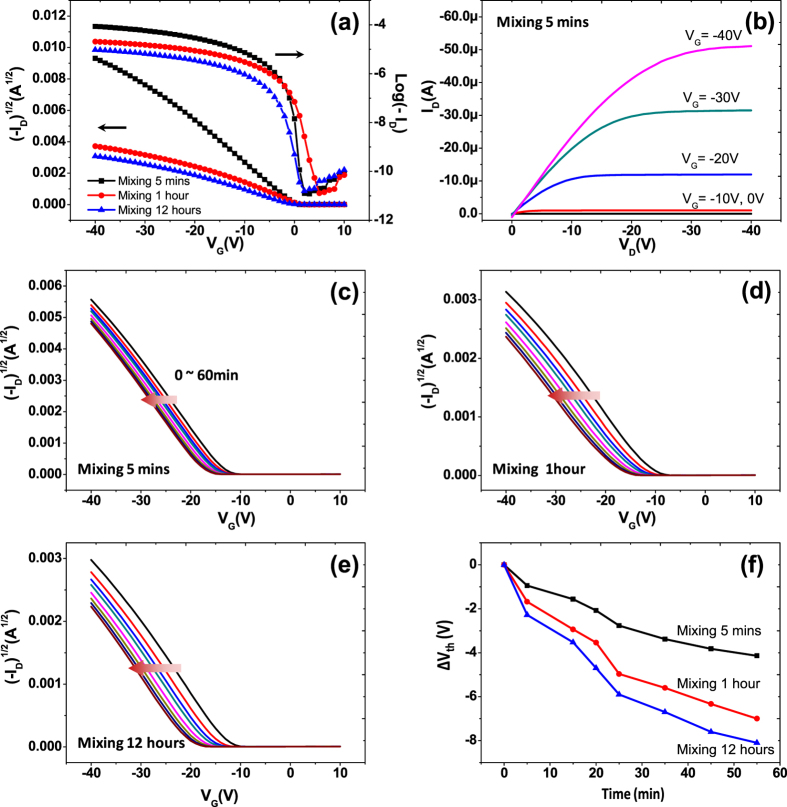
Typical transfer (**a**) and output (**b**) characteristics of TES-ADT FETs based on a PS brush-treated SiO_2_ dielectric after solvent vapor annealing. I_D_−V_G_ transfer curves of TES-ADT FETs based on a PS brush-treated SiO_2_ dielectric as a function of stress time under an applied gate bias stress of −40 V (V_D_ = −5 V) (**c**–**e**), and the relative threshold voltage shift ΔV_th_ as a function of stress time (**f**).

A polystyrene (PS) brush was used to reduce charge trapping at the semiconductor-dielectric interface while clarifying the GB-induced charge trapping^31^,^33^,^35^. PS-Si(CH_3_)_2_Cl could be grafted to the SiO_2_ surface by chemical bonding between the end-functional groups and silanol on the SiO_2_ surface, and a PS brush layer could be formed as a consequence^33^. Figure S2 in Supporting information displays polarized optical microscopy images of TES-ADT films based on PS brush-treated SiO_2_ dielectric after solvent vapor annealing. The clear differences in grain sizes among three samples can be still observed, leading to differences in electrical properties of TES-ADT FETs. There is a significant increase in the mobility after modifying SiO_2_ dielectric surfaces with PS brush. As can be seen from Fig. 6a and Table 1, the average field-effect mobility of the 5-min sample with PS brush was 0.913 cm^2^/V-s, increasing by nearly a factor of 4 compared to the 5-min sample without PS brush. The average field-effect mobilities of the 1-hour and 12-hour samples with PS brush were 2 times higher than those without PS brush. This is because of the removal of trapping sites such as silanol groups at the SiO_2_/TES-ADT interface. TES-ADT is in contact with the PS brush, which does not contain any hydroxyl groups at the surface. When the effects of the GB density were examined, GB effects with a negligible influence from the dielectric-semiconductor interface were obtained. The device with 5 mins of mixing showed the highest field-effect mobility (0.913 cm^2^/V-s), which is nearly 4 times higher than field-effect mobility of 12 hours device (0.236 cm^2^/V-s). We surmise that the reduced charge trappings at the GBs contribute to the increased field-effect mobility. In addition, the bias stability under continuous gate bias was examined by measuring V_th_ shifts (Fig. 6c–f). Because of reduced interface trapping at the SiO_2_/TES-ADT interface, devices with PS brush showed much smaller V_th_ shifts compared to those of untreated SiO_2_. The devices with large TES-ADT grain sizes (5 mins of mixing) also exhibited the highest bias stability. These results provide that both GBs in the semiconductor and the quality of semiconductor-dielectric interface can affect the electrical properties of TES-ADT FETs, such as the field-effect mobility and bias stability. In Supporting information, Figure S3 and Table S1 display electrical properties of TES-ADT FETs at low gate and source/drain biases (V_G_ = −5 V, V_D_ = −5 V) and Figure S4 shows hysteresis behavior which show similar effects of the GB densities. This result provides that GB effects are general phenomena irrespective of device operation voltage.

## Conclusions

Understanding how the microstructure of organic semiconducting layers and how GBs within these layers influence the electrical performance of OFETs is vital for the development of organic electronics. In this study, we examined the intrinsic effects of GBs on the electrical properties of TES-ADT FETs. TES-ADT solutions were prepared with mixing times ranging from 5 minutes to 12 hours. In as-spun TES-ADT thin films, many aggregates occurred when increasing the mixing time. During exposure to 1,2-dichloroethane (DCE) vapor, crystallization occurs at nucleation sites near the aggregates and grows until each spherulite meets its neighbors, resulting in TES-ADT films with different densities of GBs. The sample mixed for 5 minutes exhibited the largest grain size whereas the sample mixed for 12 hours showed the smallest grain size with the highest density of GBs. Structural characteristics showed that the angular spread and crystallographic orientations of TES-ADT spherulites were strongly affected by the density of GBs. The electrical properties such as field-effect mobility and operational stability were studied. The sample mixed for 5 minutes exhibited the highest mobility and also best electrical stability because of having the lowest density of GBs. These properties became worse when the density of GBs was increased. Additionally, PS brush was employed to modify the SiO_2_ surface. This in turn led to enhanced electrical performances compared to those of FETs with untreated SiO_2_, due to the reduction of charge trapping at the semiconductor-dielectric interface.

## Methods

### Materials and Sample Preparation

Triethylsilylethynyl anthradithiophene (TES-ADT) was synthesized according to the procedure reported by Anthony and coworkers. Heavily doped silicon wafers containing 300-nm thick SiO_2_ layer were purchased from Fine Science and were used as substrates for all samples. The wafers were then cut into pieces before cleaning by sonication in acetone and isopropyl alcohol for 20 minutes sequentially. After that, these wafers were rinsed quickly with deionized water and then dried with nitrogen gas, followed by a 10 minute UV exposure. To modify the surface of the SiO_2_ layer, dimethyl chlorosilane-terminated polystyrene (PS-Si(CH_3_)_2_Cl, M_w_ = 8000, PDI = 1.06, Polymer Source) was used. PS-Si(CH_3_)_2_Cl was mixed with toluene purchased from Aldrich Chemical Co., for 5 minutes to generate 0.3 wt% solution. The solution was then spin-coated at 1000 rpm for 30 seconds onto the pre-cleaned silicon wafers in ambient air, and the resulting films were thermally annealed at 120 °C for 30 minutes to graft PS brush onto SiO_2_/Si. The films were then sonicated in a toluene bath for 30 seconds to eliminate unreacted PS brush and dried with nitrogen gas. TES-ADT was dissolved in toluene to generate 1.5 wt% solutions and the solutions were stirred at 500 rpm for 5 minutes, 1 hour and 12 hours, respectively. Mixing was carefully done in oxygen-free/dark condition to avoid generation of the impurities. Each TES-ADT solution was then directly spin-coated onto the untreated SiO_2_/Si and PS-grafted SiO_2_/Si substrates at 1000 rpm for 60 seconds. As-spun TES-ADT films were immediately transferred to a hot plate at 90 °C to remove residual solvent. 1,2-dichloroethane (DCE) from Aldrich Chemical Co. was poured into a glass petri dish for sovent vapor annealing. The TES-ADT films were then put onto an island located in the middle of the dish before placing a cover on the top of the dish. The exposure of TES-ADT thin films to DCE solvent ended until these films became totally crystalline. The samples were then dried again in a vacuum oven. In order to fabricate top-contact FETs, gold source-drain electrodes were deposited directly on the TES-ADT films by thermal evaporation through a shadow mask with a channel length and channel width of 150 μm and 1500 μm, respectively.

### Characterization

The film morphologies were characterized using an optical microscope (Nikon). The inner structures of the TES-ADT films were characterized with 2-dimensional grazing incidence X-ray diffraction (2D-GIXD) recorded at the Pohang Accelerator Laboratory of Korea (9A and 3C beamlines). Current-voltage characteristics of all of the devices were measured using a Keithley 4200-SCS and a probe station operated under dark and vacuum conditions. After deposition of source and drain electrodes, each device was isolated by a mechanical scratch. In order to collect output characteristics, the source-drain voltage was swept from V_D_ = 0 V to V_D_ = −40 V when the gate voltage was increased from V_G_ = 0 V to V_G_ = −40 V in increments of −10 V. In order to collect transfer characteristics, the gate voltage was swept from V_G_ = 10 V to V_G_ = −40 V in increments of −1 V, while the source-drain voltage was kept unchanged at V_D_ = −40 V. Regarding bias stability, the general method for the study of bias-stress stability on organic transistors is to measure the variation in their transfer characteristics over time. A constant gate voltage and constant drain voltage (V_D_ << V_G_, linear regime) are applied to the organic transistor. In our experiments, V_G_ = −40 V and V_D_ = −5 V were applied. To determine the changes in the transfer curves, the applied gate voltage is interrupted at short time intervals by a sweep of the gate voltage. In our experiments, the gate voltage was swept from V_G_ = 10 V to V_G_ = −40 V in increments of −1 V.

## Additional Information

**How to cite this article**: Nguyen, K. V. *et al*. Grain Boundary Induced Bias Instability in Soluble Acene-Based Thin-Film Transistors. *Sci. Rep.* **6**, 33224; doi: 10.1038/srep33224 (2016).

## Supplementary Material

Supplementary Information

## Figures and Tables

**Table 1:** Electrical properties of TES-ADT transistors before and after solvent vapor annealing.

Devices	Mixing time	Mobility (cm^2^ V^−1^ s^−1^)	V_th_ (V)	I_on_/I_off_
Untreated SiO_2_ Before solvent annealing	5 mins	0.22 × 10^−2^ ± 0.0001	−10.66	1.0 × 10^5^
	1 hour	0.34 × 10^−2^ ± 0.0001	−12.67	1.0 × 10^5^
	12 hours	0.39 × 10^−2^ ± 0.0002	−11.13	1.0 × 10^5^
Untreated SiO_2_ After solvent annealing	5 mins	0.248 ± 0.013	1.64	1.0 × 10^6^
	1 hour	0.161 ± 0.022	6.98	1.0 × 10^6^
	12 hours	0.139 ± 0.010	2.50	1.0 × 10^5^
PS brush After solvent annealing	5 mins	0.913 ± 0.124	0.01	1.0 × 10^6^
	1 hour	0.358 ± 0.083	2.30	1.0 × 10^6^
	12 hours	0.236 ± 0.032	1.67	1.0 × 10^5^

**Table 2:** Extracted characteristic time (τ) and dispersion parameter (β) by fitting curves in Fig. 5f with the stretched exponential equation.

	τ [×10^4 ^s]	β
5 mins	3.33	0.60
1 hour	1.83	0.62
12 hours	0.66	0.61
