# Supplementary material for: Grain Boundary Induced Bias Instability in Soluble Acene-Based Thin-Film Transistors
Source: Sci Rep. 2016 Sep 12;6:33224. doi: 10.1038/srep33224 (PMC5018961; doi:10.1038/srep33224)
Supplement: Supplementary Information [file srep33224-s1.pdf]

# Supporting Information

**Grain Boundary Induced Bias Instability in Soluble Acene-Based Thin-Film Transistors**

Ky V. Nguyen[1], Marcia M. Payne[2], John E. Anthony[2], Junghun Lee[1], Eunjoo Song[3], Boseok Kang[3], Kilwon Cho[3], Wi Hyoung Lee[1*]

[1]Department of Organic and Nano System Engineering, Konkuk University, Seoul 05029, Korea. [2]Department of Chemistry, University of Kentucky, Lexington 40506, USA. [3]Department of Chemical Engineering, Pohang University of Science and Technology, Pohang 37673, Korea.

Correspondence and requests for materials should be addressed to W.H.L (email: whlee78@konkuk.ac.kr)

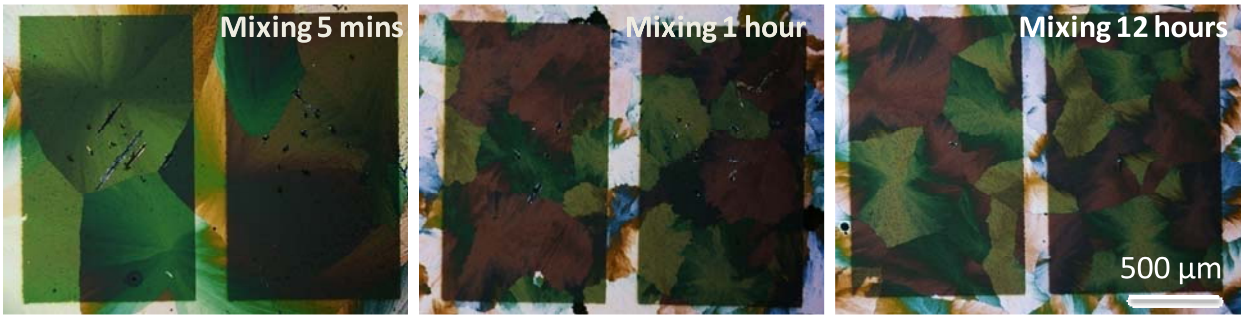


**Figure S1.** Polarized optical micrographs of the channel regions of TES-ADT FETs with different grain boundaries.

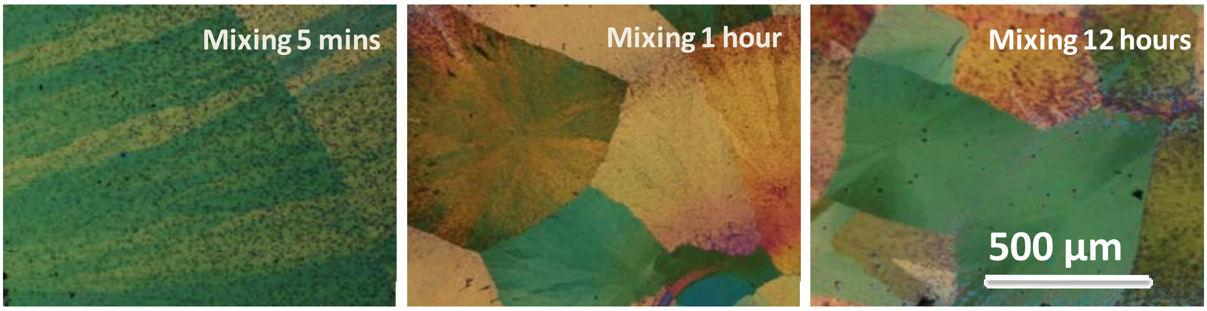


**Figure S2.** Polarized optical micrographs of TES-ADT films based on PS brush-treated $SiO_2$ dielectric after solvent vapor annealing.

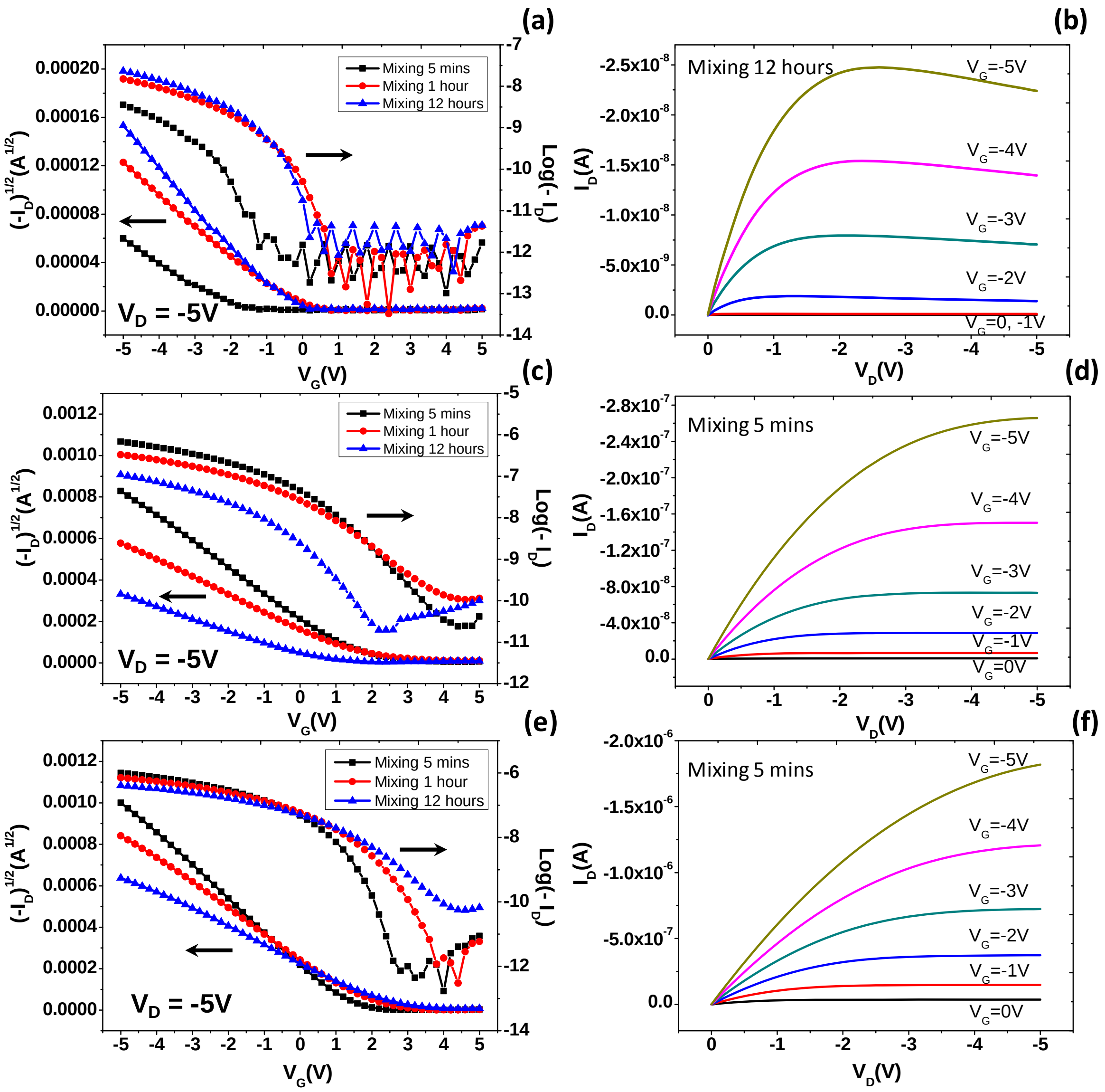


**Figure S3.** Typical transfer and output characteristics of TES-ADT FETs at low gate and source/drain biases ($V_G$ = -5 V, $V_D$ = -5 V). Before solvent annealing (a, b). An untreated $SiO_2$ dielectric after solvent vapor annealing (c, d). A PS brush-treated $SiO_2$ dielectric after solvent vapor annealing (e, f).

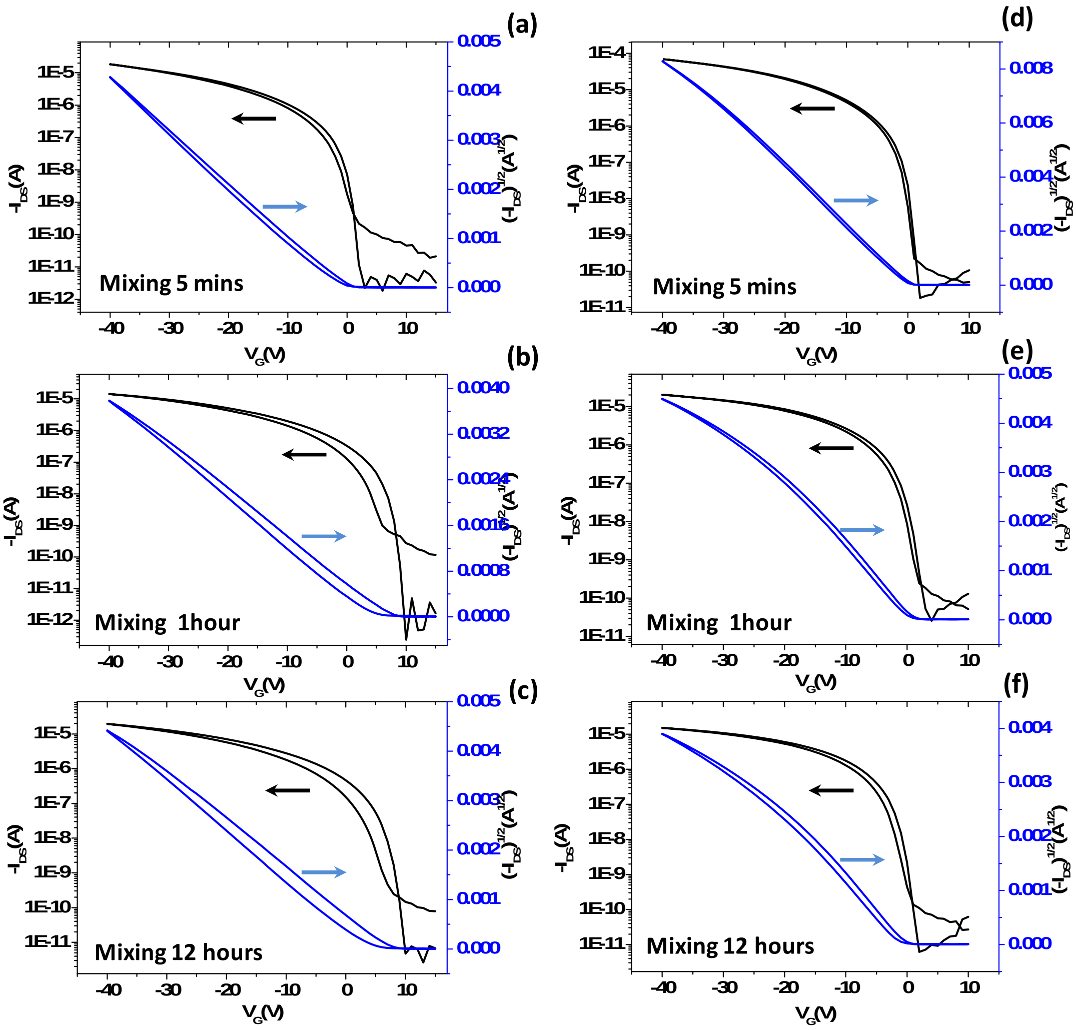


**Figure S4.** Hysteresis characteristics of TES-ADT FETs at $V_D$ = -40 V. An untreated $SiO_2$ dielectric after solvent vapor annealing (a, b, c). A PS brush-treated $SiO_2$ dielectric after solvent vapor annealing (d, e, f).

**Table S1.** Electrical properties of TES-ADT transistors before and after solvent vapor annealing at low gate and source/drain biases ($V_G$ = -5V, $V_D$ = -5V).

| Devices | Mixing time | Mobility ($cm^2V^{-1}s^{-1}$) | $V_{th}$ (V) | $I_{on}$ / $I_{off}$ |
|---|---|---|---|---|
| Untreated $SiO_2$ Before solvent annealing | 5 mins | $0.75 \times 10^{-2}$ ± 0.002 | -2.09 | $1.0 \times 10^3$ |
| | 1 hour | $1.24 \times 10^{-2}$ ± 0.0008 | -0.60 | $1.0 \times 10^4$ |
| | 12 hours | $2.15 \times 10^{-2}$ ± 0.0045 | -0.99 | $1.0 \times 10^4$ |
| Untreated $SiO_2$ After solvent annealing | 5 mins | 0.199 ± 0.019 | 1.40 | $1.0 \times 10^4$ |
| | 1 hour | 0.110 ± 0.007 | 2.32 | $1.0 \times 10^4$ |
| | 12 hours | 0.097 ± 0.010 | 0.52 | $1.0 \times 10^4$ |
| PS brush After solvent annealing | 5 mins | 0.779 ± 0.070 | 1.30 | $1.0 \times 10^6$ |
| | 1 hour | 0.308 ± 0.040 | 2.18 | $1.0 \times 10^5$ |
| | 12 hours | 0.253 ± 0.034 | 2.72 | $1.0 \times 10^4$ |
